# Application of a genetic algorithm to the keyboard layout problem

**DOI:** 10.1371/journal.pone.0226611

**Published:** 2020-01-07

**Authors:** Amir Hosein Habibi Onsorodi, Orhan Korhan

**Affiliations:** Department of Industrial Engineering, Eastern Mediterranean University, Famagusta, North Cyprus, Turkey; Bangladesh University of Engineering and Technology, BANGLADESH

## Abstract

The number of people who use computers for business and personal purposes increases as technology grows. The application of ergonomic practices on computer workstations reduces the musculoskeletal discomfort experienced and increases the overall satisfaction of the users. Keyboards are available in various systems, from computers to mobile devices, and have difference shapes and sizes. The keyboard size and shape is known to influence the user’s upper extremities. Alternative keyboard designs help diminish the pain in the arms that occurs due to awkward arm postures. Most previous studies tried to optimize the keyboard layout based on ergonomic typing and the frequency of letters’ co-occurrence. This research considers the frequency of the appearance of the most used 3,000 words in the English language. First, the frequency of each letter pair is calculated by the Text Analyzer. Then, a genetic algorithm is applied to design an ergonomically optimized keyboard to minimize the total distance of finger travel among the selected alphanumeric characters. The results showed that the distance travelled obtained by the proposed keyboard layout is less than that for the QWERTY keyboard in all different types of texts, in which an average of 6.04% improvement was achieved. Therefore, the proposed design can be used for keyboards to reduce time and fatigue.

## Introduction

The use of computers has become a necessity for all purposes. The keyboard represents one of the most popular and effective devices to insert, edit, delete and update long strings of information. Keyboards were first introduced more than 100 years ago to support the typist’s task.

Typing performance can be affected by the keyboard layout which also helps to reduce musculoskeletal disorders (MSDs). Studies show that some factors such as travel distance, amount of pressure on the key, and the tactile feedback effect muscle fatigue and discomfort [1].

QWERTY and Dvorak keyboards are the two most utilized keyboards. Since the traditional QWERTY layout appeared on the typewriter in 1878, many efforts have been made to improve its inherent inefficiency. The Dvorak Simplified Keyboard (DSK) has been very positively evaluated in terms of user performance and learnability; however, it can be very inefficient when typing in languages other than English, because it was designed using only the English corpus.

Different challenges are experienced in trying to enter information easily into the computer. Data are entered into the computer via various hardware media. Voice and handwriting are two methods for entering data, but the alphabet keyboard remains one of the best technologies for entering a large variety of information accurately and quickly.

This paper addresses the problem of text entry and proposes the optimal keyboard layout for the English language.

To correctly define this problem, the following approach is used: First, a word analysis is proposed, and then a systematic plan for the layout is applied to design a new ergonomic keyboard.

To this aim, a text analyzer was coded to analyze the words. The analyzer provides the most frequent letters and most common pairs of letters used in a text.

Thus, the main aim of this paper is to develop a new keyboard layout for the keyboard arrangement problem (KAP) that is based on the frequency of letters and to create the optimal model for a physical keyboard. It can be mentioned that only 26 letters are considered in the keyboard layout in this paper.

## Literature review

Various types of keyboards with different shapes and different layouts are currently in use. Each has various advantages and disadvantages. The QWERTY keyboard slows down the typing speed and results in unbalanced workloads for the left and right hands [2]. The QWERTY keyboard was introduced by the Sholes brothers in 1873. It was initially designed in an English context and later designed for other languages such as French and German. This gave rise to the AZERTY and QWERTZ keyboards. When compared to a random layout, the QWERTY keyboard showed that for inexperienced and relativity more experienced users, QWERTY is a superior layout [3]. Mackenzie [4] showed that typing with a QWERTY keyboard was faster than using ABC, Dvorak, Fitaly, Just Type and Telephone keyboards.

A keyboard layout has some advantages: it allows one to type text without tiredness, maximizes typing speed, reduces the number of typing errors, and allows a rapid mastery of the touch-typing method [5, 6]. In addition, the tapping workload distribution, hand and finger alternation, and hit direction should be the ergonomic criteria to be addressed to create a new keyboard [7].

The Keyboard Arrangement Problem (KAP) is known as the best potential arrangement of letters on a keyboard based on ergonomic procedures. Distinctive techniques for using an evaluation function based on a complex set of ergonomic criteria have been used to create a new layout. Table 1 provides list of the available research in the literature on the KAP.

**Table 1:** KAP research.

Research	Evaluation Function	Algorithm	Target Language
Light and Anderson, 1993	Relative Frequency of letter pair X Travel Time	Simulated annealing	English
Walker, 2003	Penalty points awarded to specific letter pairs	Genetic with two pooled approach	English
Eggers et al., 2003	Six Marsan’s ergonomic criteria	Ant Colony Optimization	French, German, English
Wagner et al., 2003	Six Marsan’s ergonomic criteria	Ant Colony Optimization	French, German, English
Deshwai and Deb, 2003	Six Marsan’s ergonomic criteria	Genetic	Hindi
Geottl et al., 2005	Four Marsan’s ergonomic criteria	Genetic	English
Malas et al., 2008	Relative Frequency of letter pair X Travel Time	Genetic	Arabic

The importance of an ergonomic design is in minimizing the travel distance, thus reducing repetitive finger activities. Suppose that a keyboard design can save 1 cm of finger travel per letter (currently approximately 5 cm per word); if a person types on average of 100 words per day (email, texting, etc.), then 100 words*5 cm*365 days is equal to 1.825 kilometers of travel that will be saved per person per year. Bi et al [8] made and studied keyboards for the English, French, Spanish, German and Chinese languages to reduce the travel distance to approximately half of QWERTY’s distance for all five languages.

Karrenbauer and Oulasvirta [9] developed a mathematical model to optimize keyboard layout. To this end, an integer programming model was proposed for the letter assignment problem and solved by the branch and bound approach. Iseri and Eksioglu [10] investigated digraph costs for keyboard layout optimization. They introduced a systematic methodology to develop ergonomic and optimal keyboard layouts. The study also investigated the effects of columns, rows, hands and periods on the digraph-tapping rate. Yang and Mali [11] studied the keyboard layout problem to reduce finger travel distance. They developed a metaheuristic simulated annealing algorithm to modify keyboard layouts. The results show that the proposed method is able to improve keyboard layouts and outperform the best one when compared with approaches reported in the literature.

## Methodology

**Fig 1:**
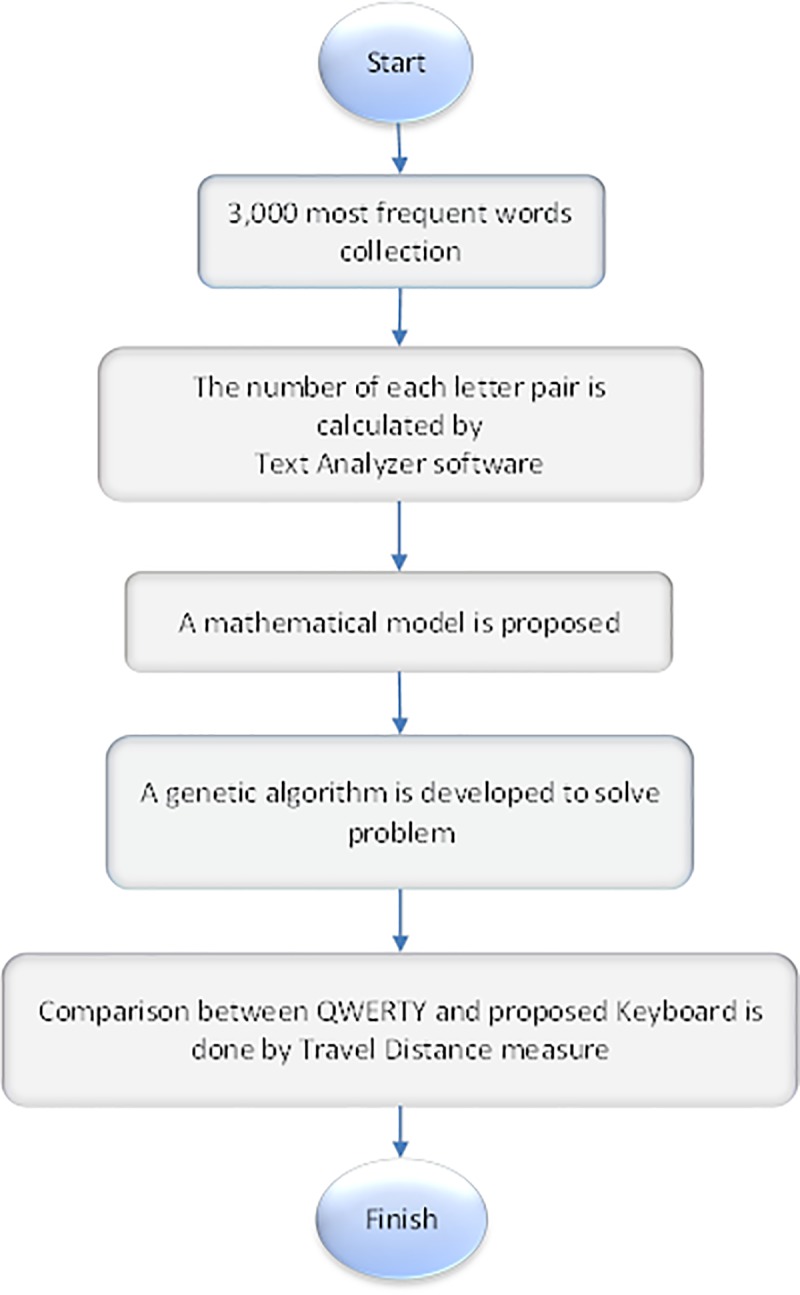
The proposed methodology.

Many research studies have been done to design a new layout for keyboards, and each of them attend to various algorithms and use different methods. Fig 1 illustrates the procedure of the proposed method. As shown in Fig 1, first, the 3,000 most common words are extracted. Next, the numbers of each pair of letters are calculated by the Text Analyzer software. Then, a mathematical programming model for the keyboard layout and a metaheuristic algorithm are developed to solve the problem. Finally, the results obtained from the proposed method are compared with the QWERTY keyboard.

However, the basis of many studies is the popularity of the letter frequency, but investigating the popularity of letter pair combinations needs more attention. Accordingly, the present study focuses on the frequency of both letter pair combinations and the popularity of each letter to design the optimal layout. The top 3,000 words that are used in daily conversation or in the analyzed text have been used.

A program is written based on the C-Sharp language to analyze words in different languages. The code first analyzes the frequency of each letter and then the pair combinations of letters. Two main factors are considered to minimize finger movement on a virtual keyboard. These two factors are the frequency of each pair of letters and the relative distances between the keys. The goal is to optimize the keyboard layout so that shortest travel distance occurs when typing on such a keyboard. This means that the most frequent keys should be located in the center of the keyboard, and the frequently connected letters should be closer to each other than are the less frequently connected letters.

**Fig 2:**
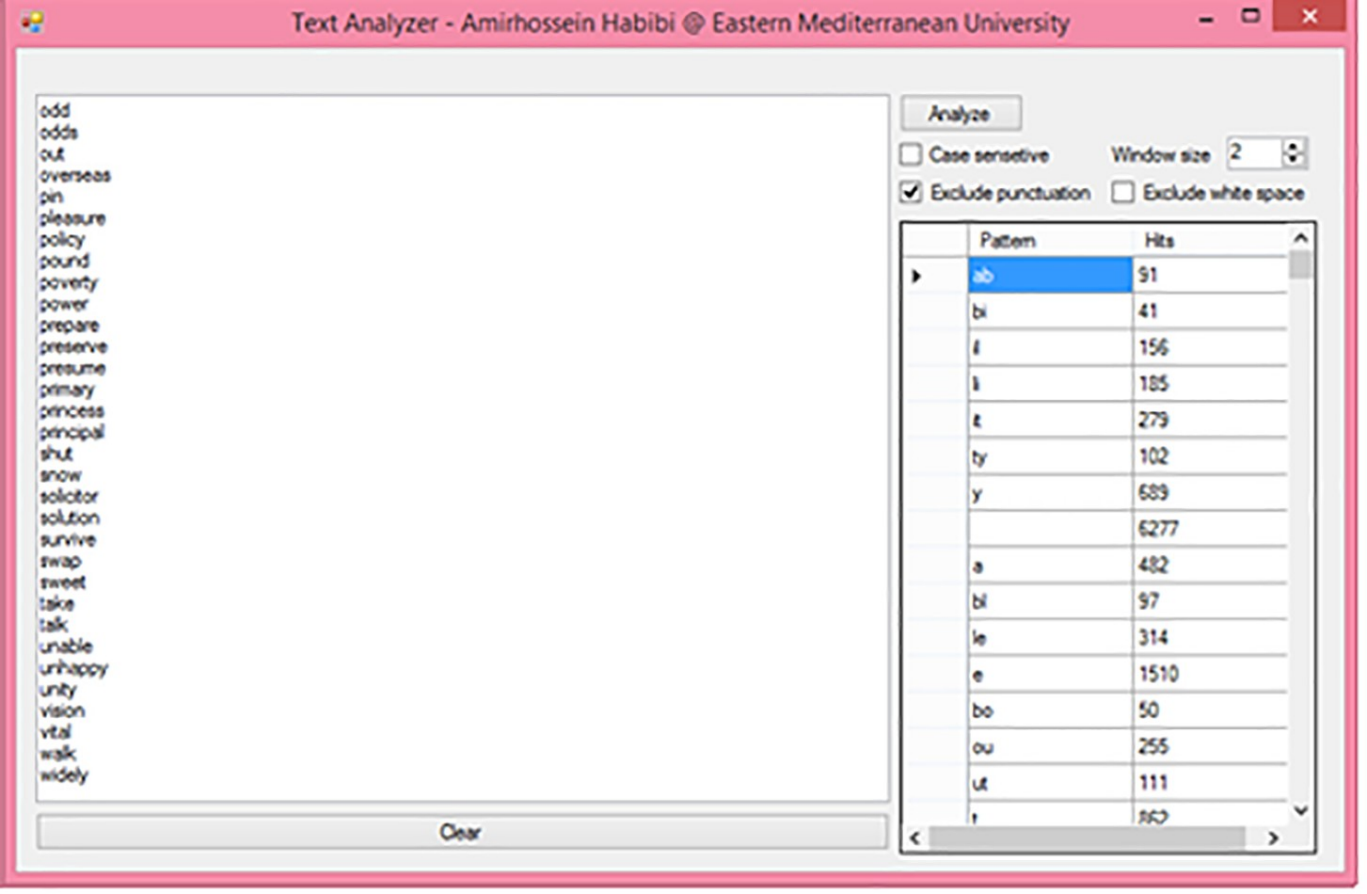
Text analyzer interface.

The interface of the program shown in Fig 2 is called the Text Analyzer. The properties of the Text Analyzer are as follows: it shows the frequency of each letter, it shows the frequency of letter combinations, it excludes or includes punctuation, it excludes or includes spaces, it is sensitive to capital letters, and it can be used for any language.

For the word selection, The Longman Communication 3,000 is used, which lists the 3,000 most frequent words in both spoken and written English based on a statistical analysis of the 390 million words contained in the Longman Corpus Network (*https://www.lextutor.ca›freq›lists_download›longman_3000_list*). An analysis of the Longman Corpus Network shows that these 3000 most frequent words account for 86% of the English language.

The program is able to count the number of each letter separately as well as the number of combinations of two or more letters. This feature is controlled by the window size option. A case sensitive option shows the difference between capital and lowercase letters. Punctuation marks such as commas, dots, question marks, etc. can even be considered.

**Fig 3:**
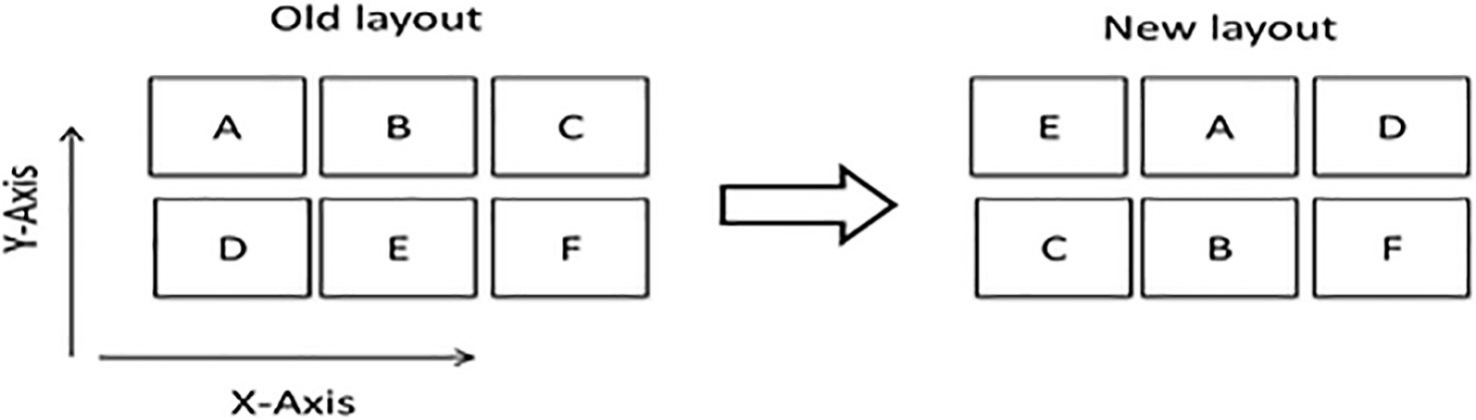
The procedure applied by the mathematical model.

It is remarkable that this program is able to be used for every language; thus, this layout designing mechanism can be developed for all languages. Therefore, dual combinations of all letters are calculated from the text analyzer (See Table A in S1 Appendix). Using the dual combinations from Table A in S1 Appendix, a mathematical programming model to design a keyboard layout is presented. Considering I, J, and K as the alias sets of keyboard letters, the X-axis and Y-axis coordination of each letter such as k (k∈K) are shown as *a*_*k*_ and *b*_*k*_, respectively. The model optimizes the existing (old) system by properly assigning each X-axis and Y-axis coordinate to the set of keyboard letters. Fig 3 represents this procedure schematically. As is illustrated in Fig 3, (2,2) represents the X-axis and Y-axis coordinates of the letter B in the existing system that is shown by *a*_*B*_ and *b*_*B*_. The model determines that in the optimal case, the coordinates of letter B, shown by *x*_*B*_ and *y*_*B*_, should be changed to (1,2). This means that the existing system shows the potential coordinates of letters, and in the next step, the model assigns the coordinates to the letters.

Since the new layout will be based on the X-axis and Y-axis coordinates of the existing system, the mathematical model assigns just one potential X-axis coordinate (such as the k^th^ coordinate) to each letter (such as i) by introducing a binary variable called *n*_*i*,*k*_, which is equal to 1 only if the k^th^ coordinate of the X-axis is assigned to i. The same mechanism is repeated by introducing a binary variable called *m*_*i*,*k*_ for determining the Y-axis coordinate of each letter i.

Sets:

i, j, and k: set of keyboard letters (i ∈ I, j∈ J, k ∈ K)

Parameters:

*w*_*i*,*j*_: the importance rate between letter i and letter j

*a*_*k*_: the X coordinate of the k^th^ key in the existing system

*b*_*k*_: the Y coordinate of the k^th^ key in the existing system

Decision Variables:

*x*_*i*_: the X coordinate of the i^th^ letter in the new system

*y*_*i*_: the Y coordinate of the i^th^ letter in the new system

$$n_{i,k}:\;\left\{ \begin{array}{l} 1\;if\;the\;k^{th}\;key\;on\;X\;axis\;is\;assigned\;to\;the\;i^{th}\;letter\\ 0\;otherwise \end{array}\right.$$

$$m_{i,k}:\;\left\{ \begin{array}{l} 1\;if\;the\;k^{th}\;key\;on\;Y\;axis\;is\;assigned\;to\;the\;i^{th}\;letter\\ 0\;otherwise \end{array}\right.$$

Model:

$$Min\;\sum_{i}\sum_{j}w_{i,j}(\left|x_{i}-x_{j}|+|y_{i}-y_{j}\right|)$$ (1)

Subject to:

$$x_{i}=\sum_{k}n_{i,k}a_{k}\;\forall i$$ (2)

$$y_{i}=\sum_{k}m_{i,k}b_{k}\;\forall i$$ (3)

$$\sum_{k}n_{i,k}=1\;\forall i$$ (4)

$$\sum_{k}m_{i,k}=1\;\forall k$$

$$\sum_{i}n_{i,k}=1\;\forall k$$ (5)

$$\sum_{i}m_{i,k}=1\;\forall k$$

The objective function minimizes the weighted distance between the keys. For each letter, constraints (2) and (3) assign a specific location on the X and Y axes. Constraint (4) guarantees that each letter is assigned to a unique location, while constraint (5) assures that each location is assigned to a unique letter. Since the mathematical model can be interpreted as a Quadratic Assignment Problem that is known to be Np-hard [12], to solve it in a large scale, a meta-heuristic algorithm is developed.

To minimize the total distance traveled on the keyboard, two components are required. The first one is the combination between each two letters, which is obtained from Table 2, and the second one is the distance between the centroid of each key on a common keyboard. That is, letter (s) next to letter (a) is one unit distant, or letter (d) is 2 units distant from letter (a). These two components are imported to the proposed model which is coded as a genetic metaheuristic algorithm in MATLAB software.

**Table 2:** Genetic algorithm parameters amount.

Symbol	Definition	Amount		
MaxIt	Maximum Number of Iterations	500	1000	1500
nPop	Population Size	1000	3000	5000
pc	Crossover Percentage	0.2	0.4	0.6
pm	Mutation Percentage	0.6	0.7	0.8
beta	Selection Pressure	4	5	6

**Fig 4:**
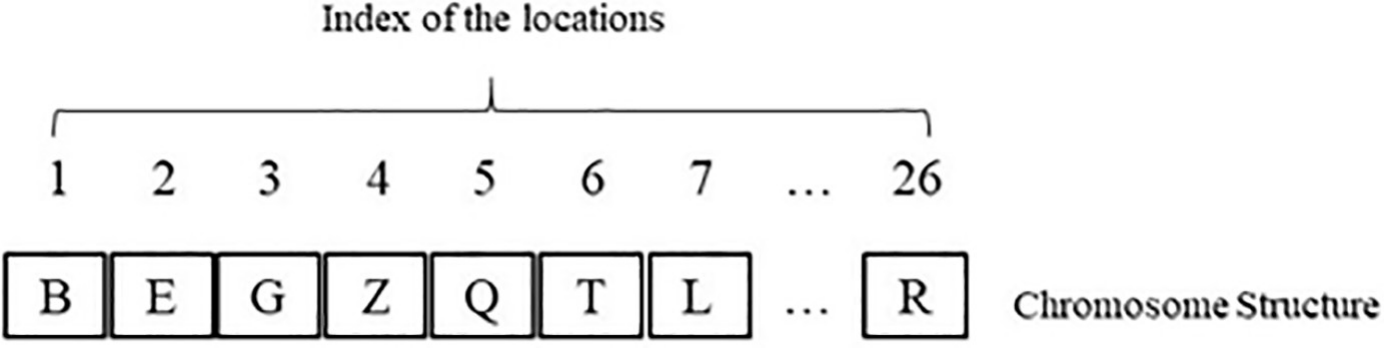
Solution encoding.

There are 26 keys and 26 designed spaces on the keyboard. The solution encoding consists of a 26-element vector. The *i*^*th*^ element determines which letter would be positioned in the *i*^*th*^ space on the keyboard. It is obvious that the distance between each pair of the spaces on the keyboard is known. The frequency of two letters used consecutively is used as the weight required for calculating the total distance (Fig 4).

**Fig 5:**
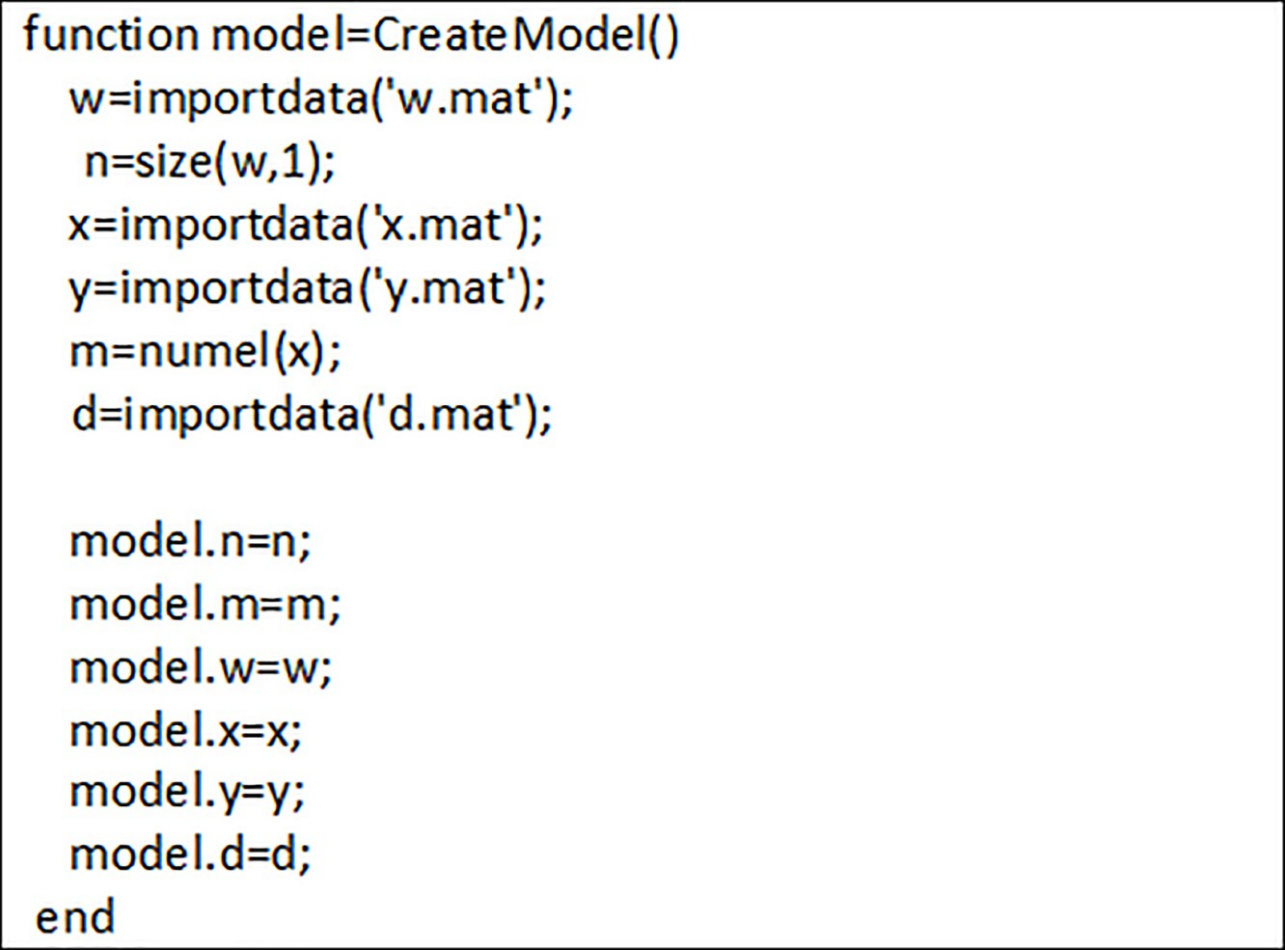
Import data.

Fig 5 shows how the required data are imported. According to this pseudo code, w is obtained from Table 2 which shows the frequency of each pair of letters. Furthermore, x and y indicate the longitudinal and transverse coordinates of each letter. Furthermore, d shows the orthogonal distance between each pair of letters. Distance between each two letter is calculated based on the distance between the centers of two letter. Tables B and C in S1 Appendix are reported w and d matrix, respectively (See S1 Appendix).

**Fig 6:**
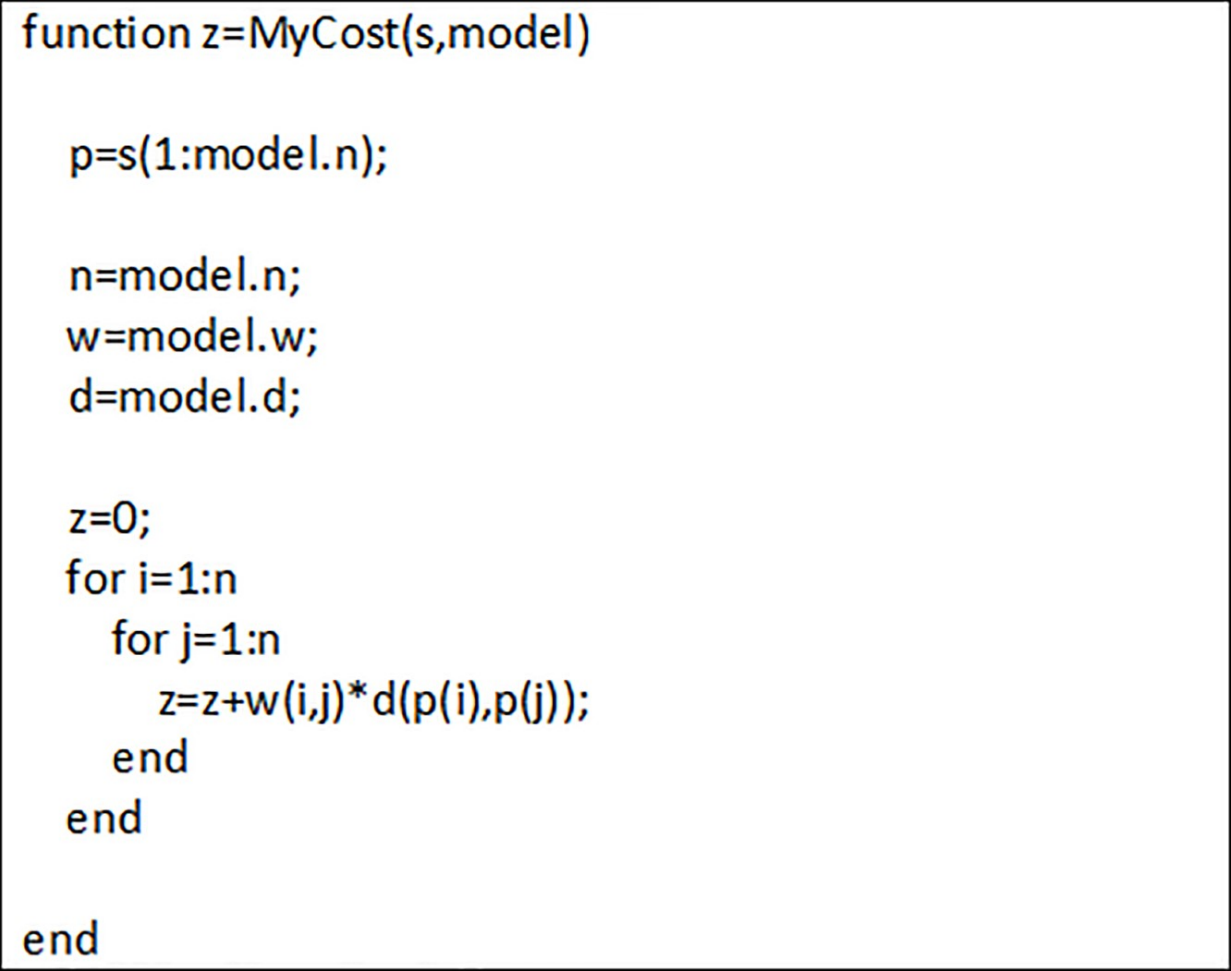
Objective function calculation.

According to these components, the proposed genetic algorithm tries to find an optimal keyboard layout so that the travel distance between letters is minimized. Based on above explanation, the procedure of calculating the objective function in the genetic algorithm is shown in Fig 6. As seen, the total travel distances are obtained from the product of the importance between each letter pair and the distance between them for all keyboards.

## Results

Genetic algorithm parameters are tuned by trial-and-error procedure. Table 2 shows the set of values for algorithm parameters on which the trials were performed.

The best value of each parameters are shown in Table 3.

**Table 3:** Best value for parameters.

Symbol	Definition	Amount
MaxIt	Maximum Number of Iterations	1000
nPop	Population Size	5000
pc	Crossover Percentage	0.4
pm	Mutation Percentage	0.8
beta	Selection Pressure	5

**Fig 7:**
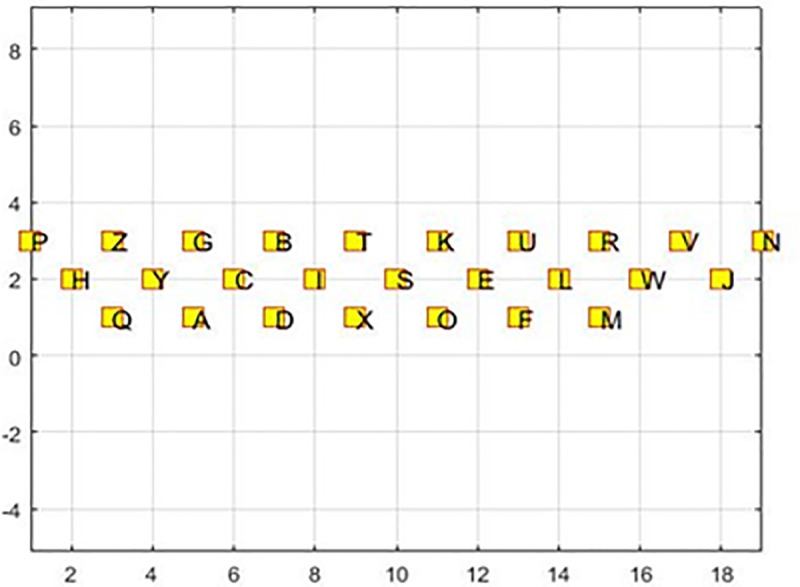
Proposed keyboard layout.

Finally, after solving the proposed genetic algorithm considering the above assumptions based on the 3000 words extracted from Longman Communication, a proposed keyboard layout is designed (Fig 7).

As shown in Fig 7, the proposed keyboard layout is different from the common QWERTY keyboard. Thus, it should be assessed as to whether the proposed layout is better than common one or not.

To validate whether the proposed keyboard layout is be better than QWERTY, 40 various texts including scientific, law, business, historical, lifestyle, study and strategy are selected. Therefore, the travel distance among the letters in each text are calculated for the QWERTY keyboard by the developed algorithm. Then, this process is repeated for the proposed keyboard. The results are reported in Table 4.

**Table 4:** Travel distance calculation for sample texts based on 2 keyboard layouts.

Type of Text	Title	Travel Distance (cm)		Reduction of Distance (%)
		QWERTY	Proposed	
Scientific	1-Astronomers think they’ve found a moon the size of Neptune in a distant star system	17,600	16,451	6.53%
	2-There's a zombie attack happening right now. It involves crickets	18,904	16,957	10.30%
	3-The ‘all-natural’ label on your LaCroix is meaningless, but that doesn’t mean it’s bad for you	19,719	18,735	4.99%
	4-Megapixels A moth drinks tears from a bird’s eye	4,044	4,006	0.94%
	5-MEGAPIXELS For a technicolor nightmare, see this fish in high definition	4,219	3,985	5.55%
Law	6-Property Law–Rights of a Tenant	16,815	16,057	4.51%
	7-Case Analysis Freedom of Speech Law	23,625	22,421	5.10%
	8-Role of Research Design in Socio-Legal Research	19,301	18,097	6.24%
	9-Doctrine of Harmonious Construction	25,977	24,227	6.74%
	10-Contract Law Case Study of Hotel	17,487	16,410	6.16%
Business	11-Critical Discussion of Corporate Social responsibility	37,629	34,307	8.83%
	12-Impacts of Nuclear Energy on Global Business	42,896	40,205	6.27%
	13-STA Travel Business Strategy	10,787	10,268	4.81%
	14-Importance of Strategic Human Resource Management	19,200	18,362	4.36%
	15-Strategic Alliances Reasons and Types	15,529	15,090	2.83%
Historical	16-18th and 21st Amendments	13,552	12,936	4.55%
	17-Missouri Compromise	11,801	11,262	4.57%
	18-Homestead Act	7,549	7,371	2.36%
	19-The Hypocrisy of American Slavery	6,947	6,662	4.10%
	20-Four Freedoms Speech	15,788	15,342	2.82%
Lifestyle	21-Sleep and cognition in children	9,762	9,109	6.69%
	22-Biological clocks and memory	11,576	10,797	6.73%
	23-Eating right for your brain	18,817	17,522	6.88%
	24-Improving attention through nature	20,833	20,088	3.58%
	25-Benefits of herbs & spices for cognition	6,676	6,289	5.80%
	26-Food & Supplements	23,312	21,364	8.36%
	27-Benefits of fruit & vegetables for cognition	6,617	6,313	4.59%
	28-Diabetes—its role in cognitive impairment and dementia	11,241	10,145	9.75%
	29-Tips for better sleep	27,906	25,075	10.14%
	30-The role of sleep in memory	22,769	21,350	6.23%
Study	31-Understanding scientific text	19,720	18,338	7.01%
	32-Reading Scientific Text	24,303	22,790	6.23%
	33-Context & the conditionalization of knowledge	23,697	22,306	5.87%
	34-Retrieval practice & the keyword mnemonic	19,195	18,429	3.99%
	35-Desirable difficulty for effective learning	17,606	16,234	7.79%
Strategy	36-Flashcards	4,716	4,811	-2.01%
	37-Subliminal & sleep learning	18,108	17,291	4.51%
	38-Interested in language	14,144	13,648	3.51%
	39-Improving attention	6,948	6,681	3.84%
	40-Similarity	8,075	7,497	7.16%
Total		665,390	625,228	6.04%

As shown in Table 4, the distances travelled obtained by the proposed keyboard layout are less than those for the QWERTY keyboard in all texts (except number 36). Therefore, the proposed design can be used for a keyboard in reality to reduce time and fatigue. Furthermore, the percentage of travel reduction in each text is reported. On average, a 6.04% improvement has been achieved.

## Discussion

The results of this study illustrate that the proposed layout provides significant improvements in typing activities. As typing is a repetitive motion, the new proposed layout for a keyboard is expected to exert less pressure on hands during typing, and thus it causes less fatigue.

Moreover, this research’s results suggest that the proposed layout of the keyboard can be used to decrease inconsistencies caused by incorrect typing.

According to Table C in S1 Appendix, the greater the number of words, the more improvement is achieved. In other words, when there are under 10000 units traveled, a 4% improvement is obtained, while a 6% improvement is achieved for more than 20000 units traveled. This shows that new keyboard layout can be used for typists who type a large amount of text every day. It helps them to type more comfortably than with the old layout.

An electromyogram study to measure the muscular activity could be considered in the future to compare the kinematics of the QWERTY layout.

## Conclusion

Even with the increasing popularity of mobile devices, many people still use computers for work and personal purposes. People who work with computers, especially those who type for long periods, are faced with ergonomic issues. One of the most important challenges in typing is that the keyboard layout affects the fatigue experienced. In this study, a new layout of a keyboard is developed to reduce the total distance traveled during the input activity.

For this purpose, first the flaws of the current keyboard layout are presented. Then, a text analyzer software was introduced to count the number of letters. The number of times that each pair of letters was put together is calculated. A mathematical program is proposed to solve the problem due to the problem’s complexity. Furthermore, a genetic algorithm is developed to achieve a new keyboard layout with a better performance.

A comparison between QWERTY and the proposed keyboard layout suggests that there has been a significant improvement in the total distance traveled using the proposed layout compared to using the QWERTY layout. In nearly each type of text for the English language, the proposed layout had an obvious superiority.

This proposed keyboard layout can be used for other Latin based languages for future research. In this study, 40 various texts are examined while much more texts can be used to validate proposed model. In order to design new layout, a genetic algorithm is used, although other approaches can be applied to develop new layout.

## Supporting information

S1 Appendix (DOCX)
