# Supplementary material for: Application of a genetic algorithm to the keyboard layout problem
Source: PLoS One. 2020 Jan 7;15(1):e0226611. doi: 10.1371/journal.pone.0226611 (PMC6946161; doi:10.1371/journal.pone.0226611)
Supplement: S1 Appendix — (DOCX) [file pone.0226611.s001.docx]

**Appendices**

Table A: Dual combinations of letters from the text analyzer

| AA | 0 | BA | 84 | CA | 142 | DA | 70 | EA | 356 | FA | 79 | GA | 59 | HA | 157 | IA | 85 |
| --- | --- | --- | --- | --- | --- | --- | --- | --- | --- | --- | --- | --- | --- | --- | --- | --- | --- |
| AB | 91 | BB | 4 | CB | 0 | DB | 4 | EB | 14 | FB | 0 | GB | 0 | HB | 2 | IB | 33 |
| AC | 207 | BC | 0 | CC | 44 | DC | 0 | EC | 245 | FC | 0 | GC | 0 | HC | 0 | IC | 254 |
| AD | 128 | BD | 0 | CD | 0 | DD | 31 | ED | 131 | FD | 0 | GD | 0 | HD | 0 | ID | 130 |
| AE | 0 | BE | 72 | CE | 320 | DE | 308 | EE | 130 | FE | 96 | GE | 161 | HE | 213 | IE | 112 |
| AF | 27 | BF | 0 | CF | 0 | DF | 2 | EF | 80 | FF | 63 | GF | 0 | HF | 0 | IF | 53 |
| AG | 103 | BG | 0 | CG | 0 | DG | 12 | EG | 48 | FG | 0 | GG | 13 | HG | 0 | IG | 119 |
| AH | 2 | BH | 0 | CH | 206 | DH | 1 | EH | 8 | FH | 0 | GH | 92 | HH | 0 | IH | 0 |
| AI | 161 | BI | 41 | CI | 124 | DI | 156 | EI | 32 | FI | 81 | GI | 50 | HI | 130 | II | 0 |
| AJ | 6 | BJ | 11 | CJ | 0 | DJ | 1 | EJ | 2 | FJ | 0 | GJ | 0 | HJ | 0 | IJ | 0 |
| AK | 39 | BK | 0 | CK | 100 | DK | 0 | EK | 8 | FK | 0 | GK | 0 | HK | 0 | IK | 20 |
| AL | 426 | BL | 97 | CL | 62 | DL | 18 | EL | 261 | FL | 16 | GL | 17 | HL | 6 | IL | 156 |
| AM | 90 | BM | 3 | CM | 0 | DM | 12 | EM | 161 | FM | 0 | GM | 0 | HM | 7 | IM | 113 |
| AN | 381 | BN | 0 | CN | 0 | DN | 2 | EN | 559 | FN | 0 | GN | 39 | HN | 8 | IN | 594 |
| AO | 2 | BO | 50 | CO | 304 | DO | 44 | EO | 14 | FO | 93 | GO | 17 | HO | 167 | IO | 367 |
| AP | 110 | BP | 0 | CP | 0 | DP | 2 | EP | 102 | FP | 0 | GP | 0 | HP | 0 | IP | 46 |
| AQ | 0 | BQ | 0 | CQ | 2 | DQ | 0 | EQ | 28 | FQ | 0 | GQ | 0 | HQ | 0 | IQ | 5 |
| AR | 426 | BR | 41 | CR | 86 | DR | 40 | ER | 707 | FR | 51 | GR | 89 | HR | 22 | IR | 119 |
| AS | 216 | BS | 16 | CS | 6 | DS | 12 | ES | 322 | FS | 0 | GS | 3 | HS | 2 | IS | 240 |
| AT | 469 | BT | 4 | CT | 219 | DT | 0 | ET | 171 | FT | 25 | GT | 7 | HT | 55 | IT | 279 |
| AU | 28 | BU | 52 | CU | 77 | DU | 68 | EU | 4 | FU | 72 | GU | 49 | HU | 28 | IU | 0 |
| AV | 44 | BV | 4 | CV | 0 | DV | 18 | EV | 102 | FV | 0 | GV | 0 | HV | 0 | IV | 106 |
| AW | 27 | BW | 0 | CW | 0 | DW | 2 | EW | 30 | FW | 2 | GW | 0 | HW | 2 | IW | 0 |
| AX | 9 | BX | 0 | CX | 0 | DX | 0 | EX | 130 | FX | 0 | GX | 0 | HX | 0 | IX | 2 |
| AY | 77 | BY | 10 | CY | 17 | DY | 25 | EY | 16 | FY | 9 | GY | 10 | HY | 11 | IY | 0 |
| AZ | 4 | BZ | 0 | CZ | 0 | DZ | 0 | EZ | 6 | FZ | 0 | GZ | 0 | HZ | 0 | IZ | 26 |
| JA | 0 | KA | 4 | LA | 181 | MA | 213 | NA | 138 | OA | 24 | PA | 174 | QA | 0 | RA | 314 |
| JB | 0 | KB | 0 | LB | 1 | MB | 23 | NB | 2 | OB | 24 | PB | 2 | QB | 0 | RB | 7 |
| JC | 0 | KC | 0 | LC | 8 | MC | 0 | NC | 202 | OC | 70 | PC | 0 | QC | 0 | RC | 57 |
| JD | 0 | KD | 0 | LD | 43 | MD | 0 | ND | 221 | OD | 50 | PD | 0 | QD | 0 | RD | 87 |
| JE | 20 | KE | 72 | LE | 314 | ME | 290 | NE | 237 | OE | 8 | PE | 242 | QE | 0 | RE | 832 |
| JF | 0 | KF | 2 | LF | 19 | MF | 1 | NF | 36 | OF | 49 | PF | 2 | QF | 0 | RF | 9 |
| JG | 0 | KG | 2 | LG | 0 | MG | 0 | NG | 240 | OG | 30 | PG | 0 | QG | 0 | RG | 48 |
| JH | 0 | KH | 0 | LH | 0 | MH | 0 | NH | 2 | OH | 1 | PH | 40 | QH | 0 | RH | 7 |
| JI | 0 | KI | 26 | LI | 185 | MI | 114 | NI | 118 | OI | 30 | PI | 60 | QI | 0 | RI | 283 |
| JJ | 0 | KJ | 0 | LJ | 0 | MJ | 0 | NJ | 9 | OJ | 3 | PJ | 0 | QJ | 0 | RJ | 4 |
| JK | 0 | KK | 0 | LK | 9 | MK | 0 | NK | 30 | OK | 23 | PK | 0 | QK | 0 | RK | 32 |
| JL | 0 | KL | 7 | LL | 223 | ML | 0 | NL | 21 | OL | 118 | PL | 122 | QL | 0 | RL | 28 |
| JM | 0 | KM | 0 | LM | 4 | MM | 42 | NM | 12 | OM | 157 | PM | 5 | QM | 0 | RM | 74 |
| JN | 0 | KN | 18 | LN | 4 | MN | 1 | NN | 31 | ON | 633 | PN | 0 | QN | 0 | RN | 60 |
| JO | 11 | KO | 2 | LO | 133 | MO | 99 | NO | 117 | OO | 77 | PO | 166 | QO | 0 | RO | 277 |
| JP | 0 | KP | 0 | LP | 9 | MP | 124 | NP | 2 | OP | 114 | PP | 70 | QP | 0 | RP | 26 |
| JQ | 0 | KQ | 0 | LQ | 0 | MQ | 0 | NQ | 2 | OQ | 0 | PQ | 0 | QQ | 0 | RQ | 0 |
| JR | 0 | KR | 0 | LR | 3 | MR | 0 | NR | 0 | OR | 345 | PR | 269 | QR | 0 | RR | 68 |
| JS | 0 | KS | 4 | LS | 11 | MS | 9 | NS | 107 | OS | 98 | PS | 14 | QS | 0 | RS | 76 |
| JT | 0 | KT | 0 | LT | 46 | MT | 0 | NT | 463 | OT | 117 | PT | 44 | QT | 0 | RT | 163 |
| JU | 5 | KU | 0 | LU | 36 | MU | 34 | NU | 30 | OU | 255 | PU | 62 | QU | 86 | RU | 62 |
| JV | 0 | KV | 0 | LV | 18 | MV | 0 | NV | 32 | OV | 78 | PV | 0 | QV | 0 | RV | 31 |
| JW | 0 | KW | 2 | LW | 5 | MW | 0 | NW | 2 | OW | 112 | PW | 0 | QW | 0 | RW | 12 |
| JX | 0 | KX | 0 | LX | 0 | MX | 0 | NX | 2 | OX | 1 | PX | 0 | QX | 0 | RX | 0 |
| JY | 0 | KY | 7 | LY | 271 | MY | 16 | NY | 33 | OY | 28 | PY | 14 | QY | 0 | RY | 120 |
| JZ | 0 | KZ | 0 | LZ | 0 | MZ | 0 | NZ | 0 | OZ | 1 | PZ | 0 | QZ | 0 | RZ | 0 |

| SA | 65 | TA | 224 | UA | 71 | VA | 54 | WA | 105 | XA | 13 | YA | 5 | ZA | 4 |
| --- | --- | --- | --- | --- | --- | --- | --- | --- | --- | --- | --- | --- | --- | --- | --- |
| SB | 3 | TB | 2 | UB | 46 | VB | 0 | WB | 2 | XB | 0 | YB | 6 | ZB | 0 |
| SC | 59 | TC | 26 | UC | 68 | VC | 0 | WC | 0 | XC | 17 | YC | 5 | ZC | 0 |
| SD | 0 | TD | 0 | UD | 43 | VD | 2 | WD | 2 | XD | 0 | YD | 0 | ZD | 0 |
| SE | 350 | TE | 499 | UE | 71 | VE | 310 | WE | 76 | XE | 7 | YE | 28 | ZE | 29 |
| SF | 15 | TF | 4 | UF | 7 | VF | 0 | WF | 3 | XF | 0 | YF | 1 | ZF | 0 |
| SG | 2 | TG | 0 | UG | 55 | VG | 0 | WG | 0 | XG | 0 | YG | 0 | ZG | 0 |
| SH | 128 | TH | 232 | UH | 0 | VH | 0 | WH | 48 | XH | 2 | YH | 1 | ZH | 0 |
| SI | 269 | TI | 517 | UI | 59 | VI | 96 | WI | 45 | XI | 16 | YI | 2 | ZI | 2 |
| SJ | 0 | TJ | 0 | UJ | 0 | VJ | 0 | WJ | 0 | XJ | 0 | YJ | 0 | ZJ | 0 |
| SK | 23 | TK | 0 | UK | 0 | VK | 0 | WK | 1 | XK | 0 | YK | 0 | ZK | 0 |
| SL | 33 | TL | 43 | UL | 114 | VL | 0 | WL | 12 | XL | 0 | YL | 0 | ZL | 0 |
| SM | 24 | TM | 16 | UM | 53 | VM | 0 | WM | 0 | XM | 0 | YM | 8 | ZM | 0 |
| SN | 5 | TN | 8 | UN | 178 | VN | 0 | WN | 23 | XN | 0 | YN | 0 | ZN | 0 |
| SO | 147 | TO | 127 | UO | 7 | VO | 23 | WO | 18 | XO | 0 | YO | 26 | ZO | 0 |
| SP | 111 | TP | 0 | UP | 40 | VP | 0 | WP | 0 | XP | 48 | YP | 5 | ZP | 0 |
| SQ | 8 | TQ | 0 | UQ | 0 | VQ | 0 | WQ | 0 | XQ | 0 | YQ | 0 | ZQ | 0 |
| SR | 1 | TR | 215 | UR | 220 | VR | 0 | WR | 0 | XR | 0 | YR | 2 | ZR | 0 |
| SS | 194 | TS | 13 | US | 152 | VS | 0 | WS | 2 | XS | 0 | YS | 26 | ZS | 0 |
| ST | 470 | TT | 65 | UT | 111 | VT | 0 | WT | 3 | XT | 29 | YT | 8 | ZT | 0 |
| SU | 150 | TU | 114 | UU | 0 | VU | 2 | WU | 0 | XU | 2 | YU | 0 | ZU | 0 |
| SV | 0 | TV | 2 | UV | 0 | VV | 0 | WV | 0 | XV | 0 | YV | 0 | ZV | 0 |
| SW | 19 | TW | 8 | UW | 0 | VW | 0 | WW | 0 | XW | 0 | YW | 5 | ZW | 0 |
| SX | 0 | TX | 0 | UX | 0 | VX | 0 | WX | 0 | XX | 0 | YX | 0 | ZX | 0 |
| SY | 17 | TY | 102 | UY | 5 | VY | 3 | WY | 0 | XY | 0 | YY | 0 | ZY | 2 |
| SZ | 0 | TZ | 0 | UZ | 0 | VZ | 0 | WZ | 0 | XZ | 0 | YZ | 0 | ZZ | 2 |

Table B: Frequency of each pair of letters (w matrix)

|  | A | B | C | D | E | F | G | H | I | J | K | L | M | N | O | P | Q | R | S | T | U | V | W | X | Y | Z |
| --- | --- | --- | --- | --- | --- | --- | --- | --- | --- | --- | --- | --- | --- | --- | --- | --- | --- | --- | --- | --- | --- | --- | --- | --- | --- | --- |
| A | 0 | 84 | 142 | 70 | 356 | 79 | 59 | 157 | 85 | 0 | 4 | 181 | 213 | 138 | 24 | 174 | 0 | 314 | 65 | 224 | 71 | 54 | 105 | 13 | 5 | 4 |
| B | 91 | 4 | 0 | 4 | 14 | 0 | 0 | 2 | 33 | 0 | 0 | 1 | 23 | 2 | 24 | 2 | 0 | 7 | 3 | 2 | 46 | 0 | 2 | 0 | 6 | 0 |
| C | 207 | 0 | 44 | 0 | 245 | 0 | 0 | 0 | 254 | 0 | 0 | 8 | 0 | 202 | 70 | 0 | 0 | 57 | 59 | 26 | 68 | 0 | 0 | 17 | 5 | 0 |
| D | 128 | 0 | 0 | 31 | 131 | 0 | 0 | 0 | 130 | 0 | 0 | 43 | 0 | 221 | 50 | 0 | 0 | 87 | 0 | 0 | 43 | 2 | 2 | 0 | 0 | 0 |
| E | 0 | 72 | 320 | 308 | 130 | 96 | 161 | 213 | 112 | 20 | 72 | 314 | 290 | 237 | 8 | 242 | 0 | 832 | 350 | 499 | 71 | 310 | 76 | 7 | 28 | 29 |
| F | 27 | 0 | 0 | 2 | 80 | 63 | 0 | 0 | 53 | 0 | 2 | 19 | 1 | 36 | 49 | 2 | 0 | 9 | 15 | 4 | 7 | 0 | 3 | 0 | 1 | 0 |
| G | 103 | 0 | 0 | 12 | 48 | 0 | 13 | 0 | 119 | 0 | 2 | 0 | 0 | 240 | 30 | 0 | 0 | 48 | 2 | 0 | 55 | 0 | 0 | 0 | 0 | 0 |
| H | 2 | 0 | 206 | 1 | 8 | 0 | 92 | 0 | 0 | 0 | 0 | 0 | 0 | 2 | 1 | 40 | 0 | 7 | 128 | 232 | 0 | 0 | 48 | 2 | 1 | 0 |
| I | 161 | 41 | 124 | 156 | 32 | 81 | 50 | 130 | 0 | 0 | 26 | 185 | 114 | 118 | 30 | 60 | 0 | 283 | 269 | 517 | 59 | 96 | 45 | 16 | 2 | 2 |
| J | 6 | 11 | 0 | 1 | 2 | 0 | 0 | 0 | 0 | 0 | 0 | 0 | 0 | 9 | 3 | 0 | 0 | 4 | 0 | 0 | 0 | 0 | 0 | 0 | 0 | 0 |
| K | 39 | 0 | 100 | 0 | 8 | 0 | 0 | 0 | 20 | 0 | 0 | 9 | 0 | 30 | 23 | 0 | 0 | 32 | 23 | 0 | 0 | 0 | 1 | 0 | 0 | 0 |
| L | 426 | 97 | 62 | 18 | 261 | 16 | 17 | 6 | 156 | 0 | 7 | 223 | 0 | 21 | 118 | 122 | 0 | 28 | 33 | 43 | 114 | 0 | 12 | 0 | 0 | 0 |
| M | 90 | 3 | 0 | 12 | 161 | 0 | 0 | 7 | 113 | 0 | 0 | 4 | 42 | 12 | 157 | 5 | 0 | 74 | 24 | 16 | 53 | 0 | 0 | 0 | 8 | 0 |
| N | 381 | 0 | 0 | 2 | 559 | 0 | 39 | 8 | 594 | 0 | 18 | 4 | 1 | 31 | 633 | 0 | 0 | 60 | 5 | 8 | 178 | 0 | 23 | 0 | 0 | 0 |
| O | 2 | 50 | 304 | 44 | 14 | 93 | 17 | 167 | 367 | 11 | 2 | 133 | 99 | 117 | 77 | 166 | 0 | 277 | 147 | 127 | 7 | 23 | 18 | 0 | 26 | 0 |
| P | 110 | 0 | 0 | 2 | 102 | 0 | 0 | 0 | 46 | 0 | 0 | 9 | 124 | 2 | 114 | 70 | 0 | 26 | 111 | 0 | 40 | 0 | 0 | 48 | 5 | 0 |
| Q | 0 | 0 | 2 | 0 | 28 | 0 | 0 | 0 | 5 | 0 | 0 | 0 | 0 | 2 | 0 | 0 | 0 | 0 | 8 | 0 | 0 | 0 | 0 | 0 | 0 | 0 |
| R | 426 | 41 | 86 | 40 | 707 | 51 | 89 | 22 | 119 | 0 | 0 | 3 | 0 | 0 | 345 | 269 | 0 | 68 | 1 | 215 | 220 | 0 | 0 | 0 | 2 | 0 |
| S | 216 | 16 | 6 | 12 | 322 | 0 | 3 | 2 | 240 | 0 | 4 | 11 | 9 | 107 | 98 | 14 | 0 | 76 | 194 | 13 | 152 | 0 | 2 | 0 | 26 | 0 |
| T | 469 | 4 | 219 | 0 | 171 | 25 | 7 | 55 | 279 | 0 | 0 | 46 | 0 | 463 | 117 | 44 | 0 | 163 | 470 | 65 | 111 | 0 | 3 | 29 | 8 | 0 |
| U | 28 | 52 | 77 | 68 | 4 | 72 | 49 | 28 | 0 | 5 | 0 | 36 | 34 | 30 | 255 | 62 | 86 | 62 | 150 | 114 | 0 | 2 | 0 | 2 | 0 | 0 |
| V | 44 | 4 | 0 | 18 | 102 | 0 | 0 | 0 | 106 | 0 | 0 | 18 | 0 | 32 | 78 | 0 | 0 | 31 | 0 | 2 | 0 | 0 | 0 | 0 | 0 | 0 |
| W | 27 | 0 | 0 | 2 | 30 | 2 | 0 | 2 | 0 | 0 | 2 | 5 | 0 | 2 | 112 | 0 | 0 | 12 | 19 | 8 | 0 | 0 | 0 | 0 | 5 | 0 |
| X | 9 | 0 | 0 | 0 | 130 | 0 | 0 | 0 | 2 | 0 | 0 | 0 | 0 | 2 | 1 | 0 | 0 | 0 | 0 | 0 | 0 | 0 | 0 | 0 | 0 | 0 |
| Y | 77 | 10 | 17 | 25 | 16 | 9 | 10 | 11 | 0 | 0 | 7 | 271 | 16 | 33 | 28 | 14 | 0 | 120 | 17 | 102 | 5 | 3 | 0 | 0 | 0 | 2 |
| Z | 4 | 0 | 0 | 0 | 6 | 0 | 0 | 0 | 26 | 0 | 0 | 0 | 0 | 0 | 1 | 0 | 0 | 0 | 0 | 0 | 0 | 0 | 0 | 0 | 0 | 2 |

Table C: Orthogonal distance between two letters (d matrix)

|  | A | B | C | D | E | F | G | H | I | J | K | L | M | N | O | P | Q | R | S | T | U | V | W | X | Y | Z |
| --- | --- | --- | --- | --- | --- | --- | --- | --- | --- | --- | --- | --- | --- | --- | --- | --- | --- | --- | --- | --- | --- | --- | --- | --- | --- | --- |
| A | 0 | 5 | 4 | 2 | 3 | 3 | 4 | 5 | 7 | 6 | 7 | 8 | 7 | 6 | 8 | 9 | 1 | 3 | 1 | 4 | 6 | 4 | 1 | 2 | 5 | 1 |
| B | 5 | 0 | 2 | 3 | 4 | 2 | 1 | 1 | 3 | 2 | 3 | 4 | 2 | 1 | 4 | 6 | 6 | 3 | 4 | 2 | 2 | 1 | 5 | 3 | 2 | 4 |
| C | 4 | 2 | 0 | 1 | 2 | 1 | 2 | 3 | 5 | 4 | 5 | 6 | 4 | 3 | 6 | 7 | 4 | 2 | 2 | 2 | 4 | 1 | 3 | 1 | 3 | 2 |
| D | 2 | 3 | 1 | 0 | 1 | 1 | 2 | 3 | 5 | 4 | 5 | 6 | 5 | 4 | 6 | 7 | 3 | 1 | 1 | 2 | 4 | 2 | 2 | 1 | 3 | 2 |
| E | 3 | 4 | 2 | 1 | 0 | 2 | 3 | 4 | 5 | 5 | 6 | 7 | 6 | 5 | 6 | 7 | 3 | 1 | 1 | 2 | 4 | 2 | 2 | 1 | 3 | 2 |
| F | 3 | 2 | 1 | 1 | 2 | 0 | 1 | 2 | 4 | 3 | 4 | 5 | 4 | 3 | 5 | 6 | 4 | 1 | 2 | 1 | 3 | 1 | 3 | 2 | 2 | 3 |
| G | 4 | 1 | 2 | 2 | 3 | 1 | 0 | 1 | 3 | 2 | 3 | 4 | 3 | 2 | 4 | 5 | 5 | 2 | 3 | 1 | 2 | 1 | 4 | 3 | 1 | 4 |
| H | 5 | 1 | 3 | 3 | 4 | 2 | 1 | 0 | 2 | 1 | 2 | 3 | 2 | 1 | 3 | 4 | 6 | 3 | 4 | 2 | 1 | 2 | 5 | 4 | 1 | 5 |
| I | 7 | 3 | 5 | 5 | 5 | 4 | 3 | 2 | 0 | 1 | 1 | 2 | 2 | 3 | 1 | 2 | 7 | 4 | 6 | 3 | 1 | 5 | 6 | 6 | 2 | 7 |
| J | 6 | 2 | 4 | 4 | 5 | 3 | 2 | 1 | 1 | 0 | 1 | 2 | 1 | 1 | 2 | 3 | 7 | 4 | 5 | 3 | 1 | 3 | 6 | 5 | 2 | 6 |
| K | 7 | 3 | 5 | 5 | 6 | 4 | 3 | 2 | 1 | 1 | 0 | 1 | 1 | 2 | 1 | 2 | 8 | 5 | 6 | 4 | 2 | 4 | 7 | 6 | 3 | 7 |
| L | 8 | 4 | 6 | 6 | 7 | 5 | 4 | 3 | 2 | 2 | 1 | 0 | 2 | 3 | 1 | 1 | 9 | 6 | 7 | 5 | 3 | 5 | 8 | 7 | 4 | 8 |
| M | 7 | 2 | 4 | 5 | 6 | 4 | 3 | 2 | 2 | 1 | 1 | 2 | 0 | 1 | 2 | 3 | 8 | 5 | 6 | 4 | 2 | 3 | 7 | 5 | 3 | 6 |
| N | 6 | 1 | 3 | 4 | 5 | 3 | 2 | 1 | 3 | 1 | 2 | 3 | 1 | 0 | 3 | 4 | 7 | 4 | 5 | 3 | 2 | 2 | 6 | 4 | 2 | 5 |
| O | 8 | 4 | 6 | 6 | 6 | 5 | 4 | 3 | 1 | 2 | 1 | 1 | 2 | 3 | 0 | 1 | 8 | 5 | 7 | 4 | 3 | 6 | 7 | 9 | 3 | 9 |
| P | 9 | 6 | 7 | 7 | 7 | 6 | 5 | 4 | 2 | 3 | 2 | 1 | 3 | 4 | 1 | 0 | 9 | 6 | 8 | 4 | 3 | 7 | 8 | 9 | 4 | 9 |
| Q | 1 | 6 | 4 | 3 | 3 | 4 | 5 | 6 | 7 | 7 | 8 | 9 | 8 | 7 | 8 | 9 | 0 | 3 | 2 | 4 | 6 | 5 | 1 | 3 | 5 | 2 |
| R | 3 | 3 | 2 | 1 | 1 | 1 | 2 | 3 | 4 | 4 | 5 | 6 | 5 | 4 | 5 | 6 | 3 | 0 | 2 | 1 | 3 | 2 | 2 | 3 | 2 | 3 |
| S | 1 | 4 | 2 | 1 | 1 | 2 | 3 | 4 | 6 | 5 | 6 | 7 | 6 | 5 | 7 | 8 | 2 | 2 | 0 | 3 | 5 | 3 | 1 | 1 | 4 | 1 |
| T | 4 | 2 | 2 | 2 | 2 | 1 | 1 | 2 | 3 | 3 | 4 | 5 | 4 | 3 | 4 | 4 | 4 | 1 | 3 | 0 | 2 | 2 | 3 | 4 | 1 | 4 |
| U | 6 | 2 | 4 | 4 | 4 | 3 | 2 | 1 | 1 | 1 | 2 | 3 | 2 | 2 | 3 | 3 | 6 | 3 | 5 | 2 | 0 | 4 | 5 | 5 | 1 | 7 |
| V | 4 | 1 | 1 | 2 | 2 | 1 | 1 | 2 | 5 | 3 | 4 | 5 | 3 | 2 | 6 | 7 | 5 | 2 | 3 | 2 | 4 | 0 | 4 | 2 | 2 | 3 |
| W | 1 | 5 | 3 | 2 | 2 | 3 | 4 | 5 | 6 | 6 | 7 | 8 | 7 | 6 | 7 | 8 | 1 | 2 | 1 | 3 | 5 | 4 | 0 | 2 | 4 | 2 |
| X | 2 | 3 | 1 | 1 | 1 | 2 | 3 | 4 | 6 | 5 | 6 | 7 | 5 | 4 | 9 | 9 | 3 | 3 | 1 | 4 | 5 | 2 | 2 | 0 | 4 | 1 |
| Y | 5 | 2 | 3 | 3 | 3 | 2 | 1 | 1 | 2 | 2 | 3 | 4 | 3 | 2 | 3 | 4 | 5 | 2 | 4 | 1 | 1 | 2 | 4 | 4 | 0 | 5 |
| Z | 1 | 4 | 2 | 2 | 2 | 3 | 4 | 5 | 7 | 6 | 7 | 8 | 6 | 5 | 9 | 9 | 2 | 3 | 1 | 4 | 7 | 3 | 2 | 1 | 5 | 0 |
